# The Immunoglobulin Superfamily Protein Differentiation of Embryonic Stem Cells 1 (Dies1) Has a Regulatory Role in Preadipocyte to Adipocyte Conversion

**DOI:** 10.1371/journal.pone.0065531

**Published:** 2013-06-17

**Authors:** Gang Ren, Cameron Beech, Cynthia M. Smas

**Affiliations:** 1 Department of Biochemistry and Cancer Biology, University of Toledo College of Medicine, Toledo, Ohio, United States of America; 2 Center for Diabetes and Endocrine Research, University of Toledo College of Medicine, Toledo, Ohio, United States of America; University College London, United Kingdom

## Abstract

Differentiation of Embryonic Stem Cells 1 (Dies1) was recently identified as a novel type I immunoglobulin (IgG) domain-containing plasma membrane protein important for effective differentiation of a murine pluripotent embryonic stem cell line. In this setting, Dies1 enhances bone morphogenetic protein 4 (BMP4) signaling. Here we show Dies1 transcript expression is induced ∼225-fold during *in vitro* adipogenesis of 3T3-L1 murine preadipocytes. Immunocytochemical imaging using ectopic expression of Flag-tagged Dies1 in 3T3-L1 adipocytes revealed localization to the adipocyte plasma membrane. Modulation of adipocyte phenotype with with tumor necrosis factor-α (TNFα) treatment or by siRNA knockdown of the master pro-adipogenic transcription factor peroxisome proliferator activated receptor gamma (PPARγ) resulted in a 90% and 60% reduction of Dies1 transcript levels, respectively. Moreover, siRNA-mediated Dies1 knockdown in 3T3-L1 preadipocytes inhibited adipogenic conversion. Such cultures had a 35% decrease in lipid content and a 45%–65% reduction in expression of key adipocyte transcripts, including that for PPARγ. The standard protocol for full *in vitro* adipogenic conversion of committed preadipocytes, such as 3T3-L1, does not include BMP4 treatment. Thus we posit the positive role of Dies1 in adipogenesis, unlike that for Dies1 in differentiation of embryonic stem cells, does not include its pro-BMP4 effects. In support of this idea, 3T3-L1 adipocytes knocked down for Dies1 did not evidence decreased phospho-Smad1 levels upon BMP4 exposure. qPCR analysis of Dies1 transcript in multiple murine and human tissues reveals high enrichment in white adipose tissue (WAT). Interestingly, we observed a 10-fold induction of Dies1 transcript in WAT of fasted *vs.* fed mice, suggesting a role for Dies1 in nutritional response of mature fat cells *in vivo*. Together our data identify Dies1 as a new differentiation-dependent adipocyte plasma membrane protein whose expression is required for effective adipogenesis and that may also play a role in regard to nutritional status in WAT.

## Introduction

White adipose tissue (WAT) is the major organ of energy storage in vertebrates, where excess energy is present as triglyceride within adipocyte lipid droplets [1], [2]. WAT is also the source of multiple adipokines that have profound impact on systemic physiology [3], [4]. The metabolic status of WAT is finely tuned for appropriate responses to the nutritional and hormonal cues of the organism. Numerous genetic and dietary murine models have illuminated the need for an appropriate mass of WAT for metabolic health. Mouse models with either reduced or excessive WAT both suffer the severe metabolic consequences of dysregulated lipid storage and metabolism. With insufficient ability to store triglyceride in WAT, either due to exceeding storage capacity as in obesity, or limited WAT storage capacity as occurs in some lipodystrophies, a lipotoxic and proinflammatory state is established [5]. Under these circumstances, free fatty acids can no longer be safely sequestered as triglyceride in the lipid droplet of white adipocytes [6]. Increased ectopic uptake and deposition of lipid occurs non-adipose cells, such as cardiac myocytes and pancreatic β cells. Non-adipocytes are ill-equipped to handle excess lipid and lipoapoptosis and other detrimental responses ensue [5].

As an appropriate mass of WAT is clearly central to a healthy metabolic state, it is therefore important to fully define the mechanisms of WAT formation and function. Over the past few decades much progress has been made in our understanding of adipogenesis, the formation of mature white adipocytes from precursors [7], [8]. Numerous classes of signaling molecules important for initiating, promoting or inhibiting this process have been identified, with much focus on transcriptional regulators [9]–[13]. While multiple transcription factors are now known to have a role in controlling adipogenesis, the steroid hormone superfamily protein peroxisome proliferator activated receptor gamma (PPARγ) is recognized as the key positive master transcription factor for adipogenesis [14], [15]. In addition to positive regulatory genes, whose expression typically increases during adipogenesis, some genes enriched in preadipocytes *vs.* adipocytes play an inhibitory function in adipogenesis [8], [16].

The 3T3-L1 preadipocyte culture model of *in vitro* adipogenesis [17]–[19] has proven extremely fruitful in identification of many adipogenesis regulators and other factors such as lipid droplet proteins and lipases that have ultimately proven key to *in vivo* adipocyte and adipose tissue development and/or function [12], [13], [20], [21]. In this highly utilized model, adipogenic conversion is initiated upon treatment of postconfluent cells with dexamethasone (Dex) and methylisobutylxanthine (MIX), generally in the presence of insulin. These components are the only exogenous factors required to propel the adipogenesis program in this culture model. The fat cells that form over the next 7–10 days possess many characteristics that define fat cells *in vivo*, such as insulin responsiveness.

Dies1, also known as VISTA and Gi24, encodes a type I transmembrane protein with a single immunoglobulin-V (Ig-V)-like domain in the extracellular region. To date, reports on Dies1 characterization and function are limited to several published studies. In 2010 Aloia and coworkers identified Dies1 during a shRNA functional screen for genes whose suppression led to inability of murine E14Tg2a pluripotent embryonic stem cells (ESCs) to undergo *in vitro* neuronal differentiation [22]. Compared to control shRNA cells, E14Tg2a ESCs with stably transfected Dies1 shRNA were also inhibited with respect to *in vitro* cardiomyocyte differentiation and yielded reduced teratoma size when injected into nude mice. Assessment of shDies1 ESCs showed persistent expression of RNA and proteins for the pluripotency markers Oct3/4 and Nanog, despite culture under differentiation-promoting conditions [22]. Additional experiments in this report indicated that Dies1 suppression blocks ESC differentiation by inhibition of bone morphogenetic protein 4 (BMP4) signaling with diminished levels of phospho-Smad1 protein observed for shRNA-Dies1 ESCs [22]. In 2012 this group found Dies1 associates with the BMP4 receptor complex in ESCs and that miR-125a targets Dies1 transcript for destruction leading to inhibition of BMP4 signaling, activation of Nodal/Activin pathways and arrest of cells in the epiblast stem cell state [23]. In a 2010 study Sakr identified Dies1, termed Gi24 in this report, during an expression cloning screen for genes whose ectopic expression in human embryonic kidney HEK293T cells promoted gelatinase activity mediated by the actions of matrix metalloprotease 2 (MMP2) and the membrane type MMP, membrane type I- matrix metalloprotease (MT1-MMP) [24], two enzymes key to extracellular matrix degradation and remodeling. Human fibrosarcoma HT1080 cells stably transfected with Dies1 had elevated expression of MT1-MMP protein at the cell surface and showed enhanced invasiveness of a collagen matrix [24]. Furthermore, transmembrane Dies1 was reported to be a target for cleavage by MT1-MMP [24]. A 2011 publication on Dies1 (termed VISTA in this report) demonstrated that expression of Dies1 on antigen presenting cells inhibited T cell proliferation and cytokine production *in vitro* [25].

In this study we used microarrays to generate transcriptional profiling data for conversion of 3T3-L1 preadipocyte to adipocytes and identified Dies1 as a new adipocyte differentiation-dependent gene. The limited published reports on Dies1 to date indicate a role in processes that can impact adipocyte and adipose tissue formation and/or function [26]. As such, we hypothesized that Dies1 might have a regulatory role in adipogenesis and/or novel functions in adipocytes. We report herein that Dies1 not only has a role in regulating adipogenesis, but that its expression is also is responsive to nutritional signals in WAT *in vivo*.

## Materials and Methods

### Cell Culture, *In Vitro* Adipocyte Differentiation and TNFα Treatment

Cell lines, with the exception of WT-BAT described below, were purchased from The American Type Culture Collection (ATCC, Manassas VA). 293T cells were cultured in Dulbecco's Modified Eagle Medium (DMEM) with 10% fetal calf serum (FCS). 3T3-L1 preadipocytes were grown in DMEM supplemented with 10% calf serum. Unless otherwise stated, chemicals for adipocyte differentiation, TNFα, BMP4, and others were from Sigma-Aldrich (St. Louis, MO).

For adipocyte differentiation 3T3-L1 preadipocytes were typically treated at 2 d post-confluence with DMEM supplemented with 10% FBS and the adipogenic inducers 0.5 mM MIX and 1 µM Dex for 48 h. Adipogenic agents were then removed, and growth of cultures continued in DMEM containing 10% FBS. In some instances 170 nM insulin was also added to differentiating cells. At five days post-induction of differentiation of naive 3T3-L1 preadipocytes, adipocyte conversion had generally occurred in approximately 90% of cells, as assessed by lipid accumulation and rounded cell morphology. For treatment of 3T3-L1 adipocytes with tumor necrosis factor alpha (TNFα) cells were incubated with or without 10 ng/ml TNFα in DMEM with 10% FCS for 24 or 72 h. Differentiation of C2C12 myoblasts to myotubes was as previously described [27].

C3H10T1/2 cells were maintained in DMEM with 10% calf serum and early passage cells were used for differentiation. For this, cells were plated at 3×10^4^ per well of 12-well plate. “Day 0” cells were harvested the next day. 2 d later cells had reached confluence and were harvested as “Day 2”. For differentiation, post-confluent cells (5 d after initial plating) were treated with DMEM supplemented with 10% FCS and 0.5 mM MIX, 1 µM Dex and 170 nM insulin for 48 h. Adipogenic agents were then removed, and cells cultured in DMEM containing 170 nM insulin and 10% FCS. “D20” cells were harvested 15 d later. At this time adipocyte differentiation had occurred in ∼90% of cells.

For differentiation of a murine brown preadipocyte cell line, referred to here as WT-BAT and obtained from C.R. Kahn (Joslin Diabetes Foundation, Harvard Medical School, Boston, MA), method was previously described [28], [29]. Briefly, cells were grown to confluence in DMEM with 10% FBS, 20 nM insulin, and 1 nM triiodothyronine. Confluent cells were incubated in this medium with 0.5 mM MIX, 0.5 µM Dex, and 0.125 mM indomethacin for 48 h. Cultures then were maintained in DMEM with 10% FBS, 20 nM insulin and 1 nM triiodothyronine.

### RNA Preparation, qPCR and Transcriptional Profiling

RNA was purified using TriZol Reagent (Life Technologies) according to manufacturer's instruction. Human WAT, testis and heart RNA was from Clontech and human liver, colon and prostate RNA from Life Technologies. For qPCR assessments, total RNA was purified from cell lysates using either an RNeasy RNA purification kit (Qiagen Corp., Valencia, CA) or a DNA-Free RNA kit (Zymo Research, Irvine, CA); in all instances a DNase I treatment step was employed. 5 µg of total RNA was typically used for first strand cDNA synthesis using SuperScript II RNase H-reverse transcriptase (Invitrogen Corp.) and an oligo(dT)-22 primer. qPCR was carried out with an ABI 7500 Real-time PCR System. Target cDNA levels were determined by SYBR green-based real-time qPCR in 25 µl reactions containing 1X SYBR or 1X Power SYBR Green Master Mix (Applied Biosystems, Foster City, CA), 400 nM forward and reverse primers, and 10 ng of cDNA. Expression was normalized against glyceraldehyde-3 phosphate dehydrogenase (Gapdh). Sequences of qPCR primers are shown in Table 1. Cycle threshold value was determined using ABI PRISM 7500 SDS software version 1.2. Dissociation plots were generated after 40 cycles and showed a single distinct sharp peak for all data presented. Analyses were performed in triplicate and statistical tests conducted using single factor ANOVA.

**Table 1:** Sequences for qPCR Primers and for siRNA Knockdown Oligonucleotides

Gene	qPCR (FOR-primer, 5′ – 3′)	qPCR (REV-primer, 5′ – 3′)
Dies1	AGGCAGGCAAAGGCTCG	CTGTCCTGCTCATTAGACGCC
GAPDH	GGCAAATTCAACGGCACAG	CGGAGATGATGACCCTTTTGG
PPARγ	GGGTGAAACTCTGGGAGATTC	TGATTCCGAAGTTGGTGGG
SCD1	TAGCTCCAGTGAGGTGGTGTG	GTGGGTTTGTTACAAGAGAAAGGATA
FABP4	TTCGATGAAATCACCGCAG	TTGTCACCATCTCGTTTTCTC
CideA	CAAACCATGACCGAAGTAGCC	AACTCCTCTGTGTCCACCAC
CideC	CAGAAGCCAACTAAGAAGATCG	TGTAGCAGTGCAGGTCATAG
ADIG	AGCAAAAGACCATCTGAGTGTG	ACCAGTGTTCTCCCTCCATC
GLUT4	AATCGCCCCCACTCATCTTC	TGCCCAGCATAGACTCCAAG
UCP1	TATCATCACCTTCCCGCTG	TGAGTCGTAGAGGCCAATC
MYOG	GCCATCCAGTACATTGAGC	GTAAGGGAGTGCAGATTGTG
BMP4	GCTACCAGGCCTTCTACTGC	ACTAGGGTCTGCACAATGGC

siRNA target	siRNA sequences (5′ – 3′)
Dies1	GACGGUACCUGCUCUCUGA CACGGUAACUUCUCUAUCA CUAAUGAGCAGGACAGUGA CCACGGAAGCCACCUGAAA
ATGL/PNPLA2	GAAAUUGGGUGACCAUCUG GGAGAGAACGUCAUCAUAU GCACAUUUAUCCCGGUGUA UGAAGCAGGUGCCAACAUU
PPARγ	CAACAGGCCUCAUGAAGAA
Non-Targeting control	UAGCGACUAAACACAUCAA

Transcriptional profiling was carried out for 3T3-L1 adipogenesis using RNA from day 0 3T3-L1 preadipocytes and day 10 3T3-L1 adipocytes. Array hybridizations were conducted for quadruplicate samples for both time points using Illumina MouseWG-6 v2.0 Expression BeadChips for mouse whole genome expression profiling of >45,200 transcripts. Following background subtraction, the average signal intensity for the quadruplicate day 0 and the quadruplicate day 10 samples was calculated. Probes for which averages were less than a value of 25 for both of the averages (Day 0 and Day 10) were excluded from consideration. Array hybridization and data analysis was on a fee-for-service basis through the University of Chicago Genomics Core at the Knapp Center for Biomedical Discovery, with array data analysis carried out by core director Dr. Pieter Faber.

### Mammalian Expression Construct for Dies1-3XFlag

A murine Dies1 expression construct, in which 3 copies of a C-terminal Flag epitope tag was fused in-frame to the Dies1 coding sequence, termed Dies1-3XFlag, was generated by PCR. We utilized a C-terminal Flag tag, as this is the same epitope tag used in studies of Dies1 in ESCs by Aloia [22]. Template DNA was a Dies1 murine cDNA clone purchased from Open Biosystems catalog #MMM1013-63099. PCR primers were based on the open reading frame of murine Dies1 GenBank sequence BC003967. Purified PCR fragment for the Dies1 open reading frame, lacking a stop codon, was cloned into the p3XFlag-CMV-14 vector (Sigma-Aldrich) and insert was fully sequence verified.

### siRNA-Mediated Knockdown Studies

siRNA was purchased from Dharmacon and unless other wised noted, was used at a final concentration of 25 nM. For Dies1, siGenome SMARTpool catalog #M-063708-01 was used. For PPARγ a single siRNA species was used, catalog #D-040712-01. For siControl (siCon), non-targeting siRNA catalog # D-001210-01-05 was used, and siGenome SMARTpool catalog # M-063708-01-0005 was used for knockdown of adipose triglyceride lipase (siATGL), the key enzyme controlling lipolysis of triglyceride in fat cells. siRNA sequences used in these studies are shown in Table 1.

To test effectiveness of Dies1 SMARTpool siRNA to knockdown Dies1 transcript, 3T3-L1 adipocytes were electroporated and RNA harvested 2 d later, cDNA synthesized and qPCR conducted. To determine Dies1 knockdown at the protein level, HEK293T cells were transfected with the Dies1-3XFlag construct with co-transfection of either control siRNA or Dies1 siRNA. Cells were harvested 48 h later and analyzed for Dies1 protein by Western blot using anti-Flag antibody. To assess effectiveness of knockdown of PPARγ protein level in siPPARγ knockdown or control siRNA knockdown 3T3-L1 adipocytes was determined by Western blot.

For studies of effect of siRNA on adipocyte differentiation, siRNA was introduced into 3T3-L1 preadipocytes using a Neon electroporation system (Invitrogen), according to manufacturer instructions using two pulses at 1400 V, with a pulse width of 20 milliseconds and utilizing 100 µl size electroporation tips. Following electroporation, cells were plated at a density of ∼5×10^5^ cells per well of 12-well plates. Cultures were confluent the next day and were treated for adipogenic differentiation with 0.25 µM Dex and 0.25 mM MIX and 86 nM insulin. The effect of Dies1 knockdown on 3T3-L1 adipocyte differentiation was tested three independent times, with triplicate cell culture wells assessed, for each specific siRNA treatment, each time; analysis was with single factor ANOVA.

For studies of effect of Dies1 knockdown on BMP4-mediated smad1 phosphorylation, 3T3-L1 adipocytes were treated with 50 nM control siRNA or siRNA for Dies1. 48 hour later, cells were serum starved for 16 h. Cells were then treated with BMP4 (50 ng/ml) or vehicle control for 15 min followed by washing and harvesting in lysis buffer containing proteinase and phosphatase inhibitors (100 mM Tris pH 7.5, 1 mM NaVO_4_, 5 mM MgCl_2_, 130 mM NaCl, 1% NP40, 1 mM EDTA, 10 mM NaF and 1X Proteinase Inhibitor (Thermo Scientific catalog #1860932)).

### Oil Red O Staining and Measurement of Intracellular Lipid Content

Cells were rinsed with phosphate buffered saline (PBS), fixed for 1 h with paraformaldehyde and neutral lipid stained with Oil Red O for 1 h using standard reagent and protocol. Cells were photographed with an Olympus IX70 and Spot Advanced software; this was also used to capture images of live cells. To document whole culture wells, a Hewlett-Packard HPC7730A scanner was used to capture digital images. Minor adjustments to images were made for better visualization. When this was done, the same brightness and contrast settings were applied to the complete figure. For quantification, Oil Red O was extracted from fixed cells with 100% isopropanol, extract diluted in 100% isopropanol and read at 490 nm using a Molecular Dynamics SpectraMax Plus384. Absorbance readings for Oil Red O stained PPARγ knockdown cultures, which contained essentially no adipocytes/lipid, were used to set background for calculation of values for siRNA control and siRNA Dies1. All O.D. readings were within the linear range for the assay, determined by measurement of serial dilutions of Oil Red O in isopropanol.

### Animal Treatments and Human Samples

This study was done in strict accord with the guidelines in the Guide for the Care and Use of Laboratory Animals of the NIH. The protocol was approved by the Institutional Animal Care and Use Committee of the University of Toledo (OLAW #A3414-01). For studies of Dies1 transcript expression in murine tissues, 8 wk old C57BL/6 male mice or *ob/ob* mice were purchased from The Jackson Laboratory.

For the dietary induced obesity (DIO) study RNA was prepared and kindly provided by Dr. K. H. Kim (Purdue University). For this study, 11 wk old ready-to-order diet induced obesity (DIO) C57BL/6J male mice and control C57BL/6J male mice were obtained from The Jackson Laboratory. In this model, mice are fed a 60 kcal% fat diet starting at 6 wks of age, with age-matched control mice fed a 10 kcal% fat diet. Mice were then maintained on the high fat or the control diet for an additional 3 wks prior to use at 14 wks of age.

For fasting and refeeding studies, 8 wk old male C57BL/6 mice were subjected to overnight (16 h) food deprivation. The next morning, animals were refed with a high-carbohydrate fat-free diet and tissue harvested 8 h later. Studies were conducted in duplicate.

Human tissue samples were purchased from indicated commercial sources as de-identified and were determined to be exempt from human subject guidelines by the University of Toledo Institutional Review Board.

### Western Blot Analysis

Cells were harvested from culture dishes by scraping into TNN (+) buffer (10 mM Tris pH 8.0, 120 mM NaCl, 0.5% NP-40, 1 mM EDTA, supplemented with a protease inhibitor cocktail). Lysates were incubated on ice for 30 min with intermittent vortexing, supernatant collected *via* centrifugation at 13,000× *g*, and protein concentration determined (Bio-Rad Laboratories). 50 µg of total protein was size-fractionated on SDS-PAGE gels and transferred onto Immobilon polyvinylidene difluoride (PVDF) membrane (Millipore Corp) for Western blot analysis. Membranes were blocked by incubation for 1 h in 5% nonfat milk/0.1% Tween 20 in PBS. This was followed by a 2 h incubation with a 1∶1000 dilution of a rabbit polyclonal anti-Flag antibody (catalog #2368, Cell Signaling Technologies), for detection of Dies1-3XFlag. For studies of BMP4-smad signaling, a 1∶2000 dilution of a rabbit monoclonal antibody for Smad1 (catalog #6944p, Cell Signaling Technologies), or that recognizes dually phosphorylated Smad1/5 (Ser463/465) (catalog #9516p, Cell Signaling Technologies), was used with overnight incubation at 4 degrees. Primary antibody incubations were followed by three 10 min washes. Washes for all Western blots were 0.1% Tween 20 in PBS. Secondary antibody was goat anti-rabbit (catalog #170-6515, Bio-Rad) at a dilution of 1∶1000 to 1∶2000 for 1 h followed by three 10 min washes. For PPARγ studies, following blocking membranes were incubated with a 1∶1000 dilution of mouse monoclonal antibody E-8 for PPARγ (catalog #sc-7273, Santa Cruz Biotechnology) for 2 h followed by three 10 min washes. Secondary antibody was 1∶1000 goat anti-mouse for 1 h followed by three 10 min washes. For Western blot loading control, a rabbit polyclonal antibody for peptidylprolyl isomerase A (PPIA)/cyclophilin A (catalog #07-313, Millipore Corporation) was used at a 1∶1000 dilution. Signals were detected with ECL Plus enhanced chemiluminescence (GE Healthcare) and digital images captured with an Alpha Innotech FluorChem HD Imaging System. Western blot images derived from distinct antibodies are demarcated by a black horizontal line. Minor adjustments of brightness and contrast were carried out to better visualize data with the same adjustments applied to the complete image panel as a whole.

### Immunocytochemistry and Confocal Imaging

The Dies1-3XFlag expression construct was introduced into differentiated 3T3-L1 adipocytes using a Neon electroporation system (Life Technologies). Following electroporation cells were plated onto laminin-coated coverslips. At 48 h post-transfection, cells were fixed with 4% paraformaldehyde and permeabilized using 0.2% Triton X-100. After blocking with 0.1% bovine serum albumin (BSA) for 30 min, rabbit polyclonal anti-Flag antibody (catalog #2368, Cell Signaling Technologies) was used as primary antibody at a 1∶200 dilution and Alexa Fluor 546 goat anti-rabbit IgG secondary antibody (catalog #A-11035, Life Technologies) was at a 1∶600 dilution. Antibody incubations were followed by three 10 min washes in PBS. For visualization of lipid droplets, neutral lipid was stained with dipyrrometheneboron difluoride (BODIPY) 493/503 (catalog #D-3922, Life Technologies) and nuclei were stained with 10 µM 4′,6-diamidino-2-phenylindole (DAPI) for 10 min. Negative controls, which yielded essentially no signal, included staining with primary antibody only, secondary antibody only, and vector-only transfectants. After processing, coverslips were mounted and imaged by confocal microscopy using the resources of the Advanced Microscopy and Imaging Center at the University of Toledo Health Science Campus. Images were captured with a Leica TCS SP5 broadband confocal microscope (Leica, Mannheim, Germany) equipped with Argon-488 and diode-pumped solid-state-561 laser sources and 63.0×1.40 N.A. oil immersion objective. Optical Z sections, 0.5 µM in thickness and totaling 5–6 µM, were collected. Laser intensities and microscope settings between samples were kept constant.

### Lipolysis Assay

3T3-L1 adipocytes were transfected with siRNA for Dies1, ATGL or a control non-targeting siRNA (siCon) using Dharmafect reagent. At 48 h post-transfection, culture media was changed to serum-free DMEM lacking phenol red. To assess basal or hormone-stimulated lipolysis, adipocytes were then incubated with or without 1 µM isoproterenol for 3 h or 12 h. Media was harvested, centrifuged for 10 min at and 1000× *g*, and supernatant collected. 20 µl of media was mixed with 200 µl of Sigma free glycerol reagent (Sigma-Aldrich, catalog # F6428) to determine glycerol content in media. Transfections of indicated siRNAs were conducted in at least quadruplicate wells and glycerol assay done in duplicate *per* sample well. The overall experiment was conducted twice with essentially the same results; one such study is presented. siRNA sequences used in these studies are shown in Table 1. Means of the values for the duplicate assay wells were used to calculate group means (*n = 6*) for the siDies1, siATGL and siCon and group means were compared for statistical significance using single factor ANOVA.

### Statistical Assessment

Single factor ANOVA was utilized with a value of p<0.05 indicating statistical significance. Additional details regarding experimental design and data analysis are presented in specific methods sections and/or respective figure legends.

## Results

### Dies1 Transcript Expression is Increased during Adipogenesis and is Highly Enriched in WAT

To ascertain gene expression changes in adipogenesis we conducted transcriptional profiling of 3T3-L1 adipogenesis by assessing preadipocytes (day 0) and day 10 mature adipocytes, generated by standard adipogenic induction protocol. Quadruplicate time point samples were analyzed on whole murine transcriptome Illumina arrays and differential gene expression determined for day 0 *vs.* day 10. Inspection of these data for previously unstudied genes that were induced during adipocyte differentiation led us to identify a novel immunoglobulin domain containing gene whose official gene symbol is 4632428N05Rik; the human Dies1 gene is designated C10orf54. Microarray data showed a ∼350-fold induction of 4632428N05Rik during adipogenesis. While our studies were underway, the 4632428N05Rik gene was reported in several other studies as Dies1, VISTA or Gi24. Other than the Riken numerical designation, there is not yet a formal gene name/symbol for this gene. Since our studies also describe the role of this gene in differentiation, we will refer to it herein as Differentiation of Embryonic Stem Cells 1 (Dies1).

**Figure 1:**
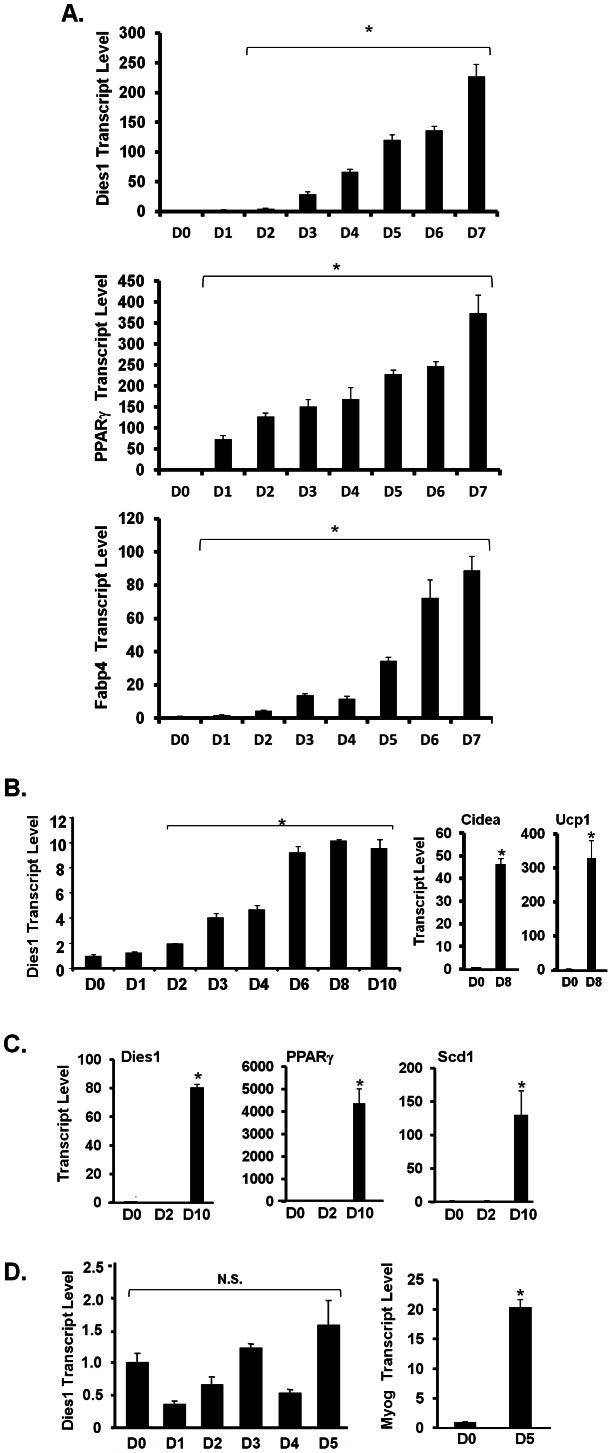
Dies1 Transcript Expression during *in vitro* Adipogenesis. RNA was purified at indicated time points (D, days) during a differentiation time course and levels of Dies1 or other indicated transcripts determined by qPCR for: ***A. 3T3-L1 adipogenesis;*** ** ***B. WT-BAT brown adipogenesis; C. CH310T1/2 mesenchymal stem cell adipogenesis; D. C2C12 myogenesis.*** For ***D***, left panel shows Dies1 transcript and the right panel transcript for the myogenesis marker myogenin (Myog). For ***A–D***, the labeling of X-axis refers to days post induction of differentiation, with D0 indicating preadipocytes, except in the case of ***D***, where D0 indicates C2C12 myoblasts. * indicates *p*<0.05, compared with the mean level at each of the D0 time points, which are set to a value of 1. The grouped data bars in ***A*** and ***B*** indicate statistical significance for each individual bar compared to the D0 value. For the left panel of ***D***, N.S. indicates not-significant (*p*>0.05) for D5 *vs.* D0. Differentiation studies were conducted twice.

The upper graph of Figure 1A shows expression of Dies1 transcript during a 7 d time course of differentiation of 3T3-L1 preadipocytes to adipocytes. Dies1 transcript is significantly increased by ∼25-fold at 3 d of adipocyte differentiation and continues to further increase thereafter. A maximal of ∼225-fold increase is achieved when the mature fat cell phenotype is uniformly attained at 7 d. This magnitude of increase is consistent with our microarray data. The lower two graphs in Figure 1A shows expression of the adipogenesis marker transcripts PPARγ and adipocyte fatty acid binding protein 4 (Fabp4); the dramatic increase in these two transcripts attests to effective adipogenesis in these samples. We also examined Dies1 expression during *in vitro* conversion of a brown preadipocyte cell line, Figure 1B. While Dies1 transcript is also upregulated during brown adipogenesis, the degree of increase is more moderate (∼10-fold) than that observed for adipocyte differentiation of 3T3-L1 preadipocytes. The two panels on the right in Figure 1B, show data for samples for preadipocyte (D0) and adipocyte (adi, D8) levels of two markers of brown adipocyte conversion which are specifically expressed in brown adipocytes, cell death-inducing DFFA-like effector a (Cidea) and mitochondrial uncoupling protein 1 (Ucp1). Cidea is a lipid droplet associated protein important in brown fat metabolism and Ucp1 action is responsible for the thermogenic regulation of brown fat. The massive increase in these two transcripts attests to effective brown adipogenesis in this study. In addition to these two models of conversion of committed preadipocytes to adipocytes, we also assessed expression of Dies1 in adipogenic differentiation of mesenchymal stem cells using C3H10T1/2 cells, and observed a ∼80-fold increase in Dies1 transcript level during adipogenesis, shown in the left panel of Figure 1C. The middle and right panel of Figure 1C show expression of the adipocyte marker transcripts PPARγ and stearoyl-Co-A desaturase 1 (SCD1), an enzyme functioning in fatty acid synthesis in these samples and confirm robust adipogenesis for the C3H10T1/2 cultures. Lastly, because Dies1 has been shown to play a role in differentiation of ESCs to several lineages, we addressed whether increased levels of Dies1 transcript occurred with *in vitro* differentiation of C2C12 committed myoblasts to myocytes. Unlike the striking increase in Dies1 transcript observed with adipocyte conversion of preadipocytes, Figure 1D shows that Dies1 transcript expression did not appreciably change during *in vitro* myogenesis. Upregulation of myogenin transcript (right panel) attests to effective myocyte differentiation of these cells, as did morphological phenotype of multinucleated aligned myotubes (data not shown).

### Dies1 Localizes to the Adipocyte Plasma Membrane

**Figure 2:**
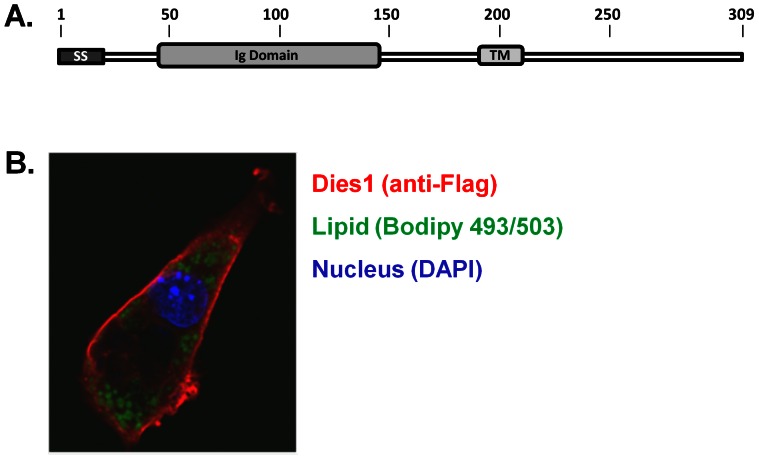
Localization of Dies1 Protein in 3T3-L1 Adipocytes. ***A. Dies1 protein domains.*** Numbers indicate AA positions for murine Dies1. SS, signal sequence; Ig, Immunoglobulin type domain; TM, transmembrane domain. ***B. Localization of Dies1 protein in adipocytes.*** 3T3-L1 day 5 adipocytes were electroporated with the Dies1-3XFlag expression construct and immunocytochemical detection carried out 48 h later. Red signal is Dies1 stained with anti-Flag antibody, lipid is stained green with Bodipy 493/503, and DAPI staining of nucleus appears blue. A representative individual Z-section from two independent imaging studies is shown.

A schematic depiction of Dies1 protein structure is shown in Figure 2A. Murine Dies1 protein is comprised of 309 amino acids (AA). As originally described by Aloia [22], Dies1 is comprised of a predicted N-terminal signal sequence, a 136 AA Ig-V domain followed by a 23 AA stalk region, a single transmembrane region and a 97 AA cytoplasmic region. B.L.A.S.T. search shows that the cytoplasmic region appears to be unique with no apparent protein domains nor homologies to other proteins. Based on ectopic expression studies in COS cells, murine Dies1 was demonstrated to localize to plasma membrane [22]. However, to our knowledge localization of Dies1 protein in a cell type(s) positive for endogenous Dies1 expression have not been conducted. As such, we determined if Dies1 localized to the adipocyte cell surface. For this we utilized transient transfection of day 5 3T3-L1 adipocytes with the Dies1-3XFlag expression construct. At 48 h post-transfection, adipocytes were fixed and immunocytochemical staining performed with an anti-Flag primary antibody and an AlexaFluor 546-conjugated secondary antibody. DAPI was used for nuclear staining, and lipid droplets visualized with Bodipy 493/503 staining. As shown by the confocal image in Figure 2B, red signal for Dies1 is clearly enriched at the cell periphery, consistent with localization to the adipocyte plasma membrane.

### Negative Regulation of Dies1 Transcript Level by TNFα

**Figure 3:**
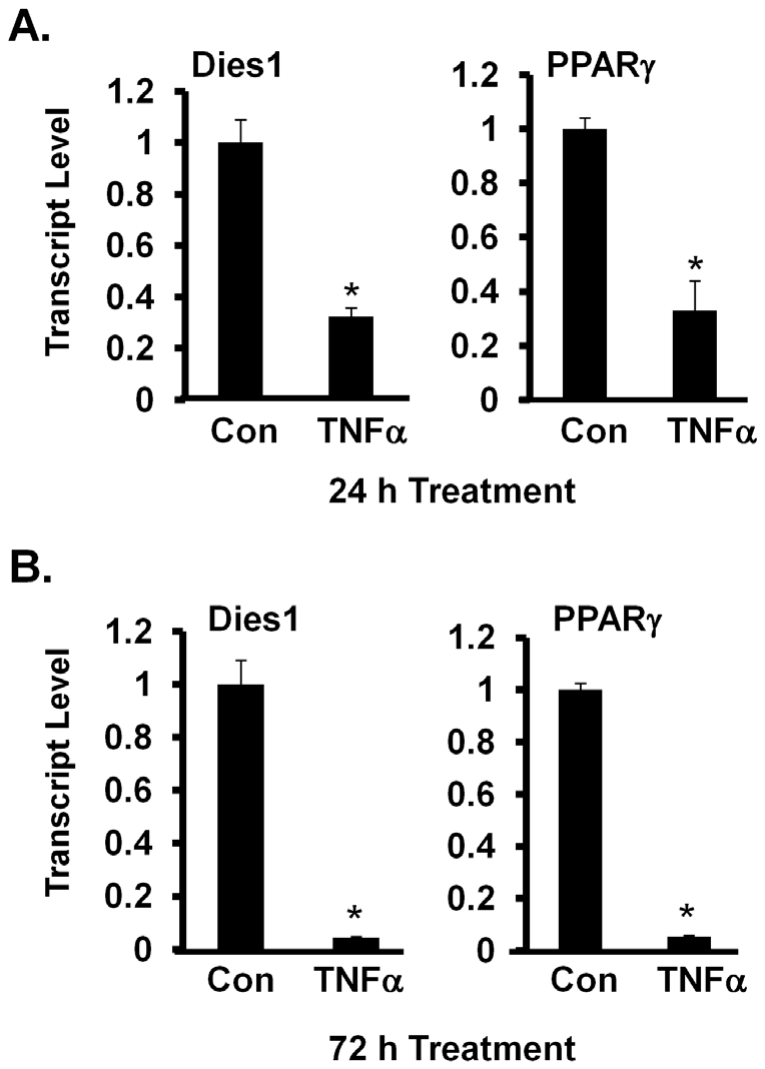
Down-regulation of Dies1 Transcript in TNFα-Treated Adipocytes. ***A. 24 h TNFα treatment.*** 3T3-L1 adipocytes were treated for 24 h with either vehicle (Con) or 10 ng/ml TNFα Transcript levels for Dies1 (left panel) and PPARγ (right panel) was determined by qPCR. ***B. 72 h TNFα treatment.*** Treatment and analysis for Dies1 (left panel) and PPARγ (right panel) transcript was as for ***A***. For ***A*** and ***B***, * indicates *p*<0.05, with respective control values set to 1. One of two representative analyses is shown.

We next employed TNFα as a way to manipulate adipocyte phenotype and function and assessed the effects on Dies1 level. TNFα is well-described to modulate phenotypic and molecular changes in fat cells to stimulate lipolysis and an adipocyte de-differentiation response. Such cells lose lipid content and convert from the rounded shape of a mature fat cell to a fibroblastic appearance that typifies preadipocytes. Study of this process at the transcript level has revealed that adipocytes treated with TNFα markedly downregulate a large subset of genes, including PPARγ that are typically induced during preadipocyte to adipocyte conversion [30], [31]. On the other hand, TNFα-treated fat cells express elevated levels of transcripts for genes typically enriched in preadipocytes *vs.* adipocytes, such as an array of extracellular matrix (ECM) components [30]–[32]. Figure 3A and 3B show Dies1 transcript levels in 3T3-L1 adipocytes are reduced by 65% and 95% with a respective 24 h and 72 h exposure to 10 ng/ml TNFα. We also measured PPARγ transcript levels, which are well established to be reduced upon TNFα treatment [33]. As anticipated, PPARγ transcript level is markedly diminished at 24 h and 72 h of TNFα treatment.

### Reduced Dies1 Transcript Level in Adipocytes Knocked Down for PPARγ

**Figure 4:**
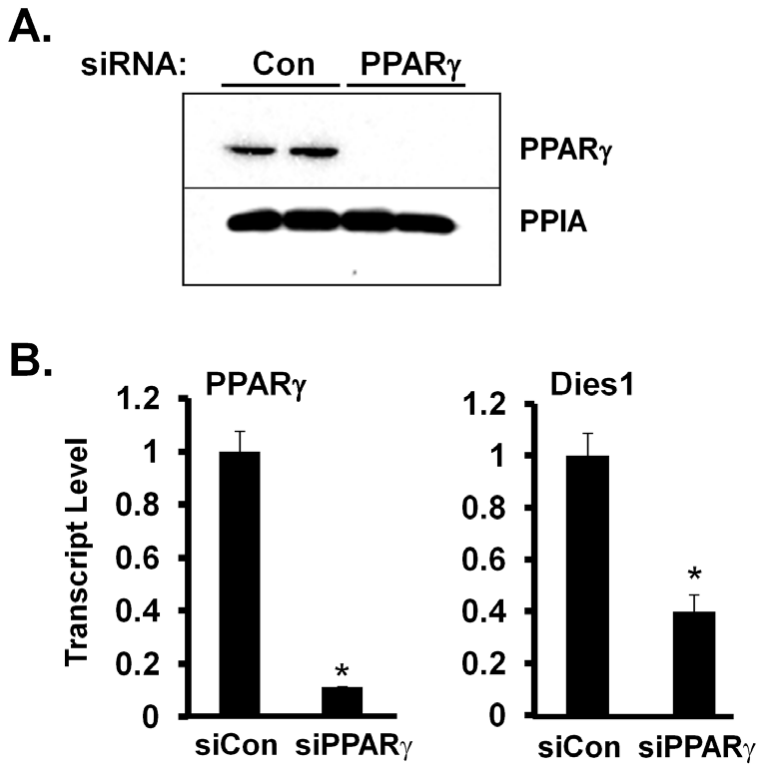
siRNA-Mediated Knockdown of Adipocyte PPARγ Decreases Dies1 Transcript Level. ***A. Effectiveness of siRNA-mediated knockdown of endogenous PPARγ protein in 3T3-L1 adipocytes.*** Control (Con) or PPARγ siRNA was introduced into day 14 3T3-L1 adipocytes. 2 days later total protein was harvested and analyzed by Western blot for PPARγ or PPIA, with the latter serving as a loading control. ***B. Response of PPARγ and Dies1 transcript to PPARγ knockdown.*** PPARγ (left panel) and Dies1 transcript (right panel) levels were measured by qPCR. * indicates *p*<0.05 compared to siCon, with the value of siCon set to 1. One of two representative analyses is shown.

PPARγ agonists have been effective in treatment of diabetes, with some of the effects of such drugs due to actions on fat cells. Thus identifying genes under the direct or indirect control of PPARγ in fat cells may ultimately lead to identification of additional therapeutic treatment targets. As shown in Figure 4A, siRNA knockdown of PPARγ in *in vitro* differentiated day 14 3T3-L1 adipocytes results in a dramatic decrease in level of PPARγ protein at 48 h post-knockdown. The effect of PPARγ knockdown on PPARγ transcript level is in the left panel of Figure 4B. As shown in right panel of this figure, at 48 h after knockdown of PPARγ in adipocytes, Dies1 transcript level had decreased by 60%. This indicates that expression of Dies1 transcript in fat cells is dependent, at least in part, on PPARγ-mediated signals.

### siRNA-Mediated Silencing of Dies1 Inhibits Adipogenesis

**Figure 5:**
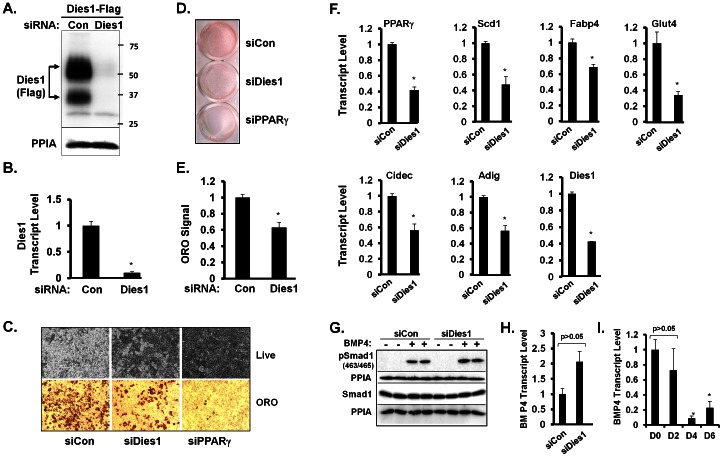
siRNA-Mediated Knockdown of Dies1 Inhibits Adipogenesis. ***A. Effectiveness of siRNA for Dies1 protein knockdown.*** Dies1-3XFlag expression construct was transiently transfected into 293T cells along with either a control siRNA (Con) or siRNA for Dies1. Total protein was harvested 48 h later. Western blot with a Flag antibody was conducted on cell lysates with PPIA as loading control. Numbers on right indicate molecular mass markers in kDa. Two prominent species of Dies1 protein are detected (arrows), which represent products of post-translation modification by glycosylation [22]. ***B. Effectiveness of siRNA for knockdown of endogenous Dies1 transcript.*** Control (Con) siRNA or siRNA for Dies1 was introduced into 3T3-L1 adipocytes. RNA was harvested 48 h later and transcript levels for Dies1 measured by qPCR. * indicates *p*<0.05 compared to siCon, the value of which was set to 1. Studies in ***5A*** and ***5B*** were conducted in duplicate. ***C. Effect of Dies1 knockdown on triglyceride content during 3T3-L1 adipogenesis.*** 40× microscopic view of day 7 adipocytes post knockdown with siCon, siDies1 or siPPARγ. Following electroporation of indicated siRNAs into 3T3-L1 preadipocytes, cells were then subject to an adipogenic differentiation protocol. At 7 d post adipogenic induction, cells were either photographed live (upper panels) or following Oil Red O (ORO) staining of fixed cells (lower panels). ***D. View of complete culture wells stained with Oil Red O.*** Experiment was conducted as in ***C***. Cells harboring indicated siRNA were fixed with paraformaldehyde and stained with Oil Red O. ***E. Quantification of adipocyte neutral lipid content.*** ORO was extracted from stained culture dishes and quantified by measuring absorbance at O.D. 490 nm. *n* = 3 wells for each of siCon and siDies1. Data shown in ***5C***, ***5D***, and ***5E*** is representative of 3 independent experiments. * indicates *p*<0.05 compared to siCon, which is set to a value of 1. ***F. Assessment of levels of adipocyte marker transcripts.*** RNA was harvested from cultures of day 7 3T3-L1 adipocytes that had been subject to transfection with siCon or siDies1, as described in ***C***. qPCR analysis was conducted for the indicated transcripts and are representative of 3 independent experiments. * indicates *p*<0.05 compared to siCon, which is set to a value of 1. ***G. Knockdown of Dies1 during 3T3-L1 adipogenesis does not abrogate BMP4-induced levels of phospho-smad1*** **.** As described in materials and methods, 3T3-L1 cells were subject to siRNA-mediated knockdown utilizing siCon or siDies1. Two days post-knockdown, cultures were treated with BMP4 (+) or vehicle (−) for 15 minutes, and cell lysates analyzed for levels of pSmad1(Ser463/465)and total Smad1by western blot. PPIA signals are shown directly below each respective panel. ***H. qPCR for BMP4 transcript expression in 3T3-L1 cultures transfected with either siCon or siDies1.*** The samples are the same utilized in data shown in Figure ***5F***. ***I. qPCR for BMP4 transcript expression during 3T3-L1 adipogenesis.*** X-axis shows days (D) post-induction of adipocyte differentiation. * indicates *p*<0.05 compared to the D0 and D2 samples. Studies in ***5G***, ***5H*** and ***5I*** were done in duplicate.

To determine if Dies1 has a role in regulating adipogenesis, we conducted knockdown studies by treating 3T3-L1 preadipocytes with siRNA for Dies1 (siDies1) or control siRNA (siCon) and subjecting cells to adipogenic differentiation. Seven days post-induction of differentiation, neutral lipid content was determined by Oil Red O staining and transcript levels of key markers of adipocyte differentiation and/or mature adipocytes measured by qPCR. We used siPPARγ treatment of 3T3-L1 preadipocytes as a positive control, as PPARγ is requisite for adipogenesis. We first assessed the efficacy of siRNA for murine Dies1 at the protein and transcript level. We have been unable to identify a good commercially available antibody for endogenous Dies1. As such, we assessed effects of siRNA-mediated Dies1 knockdown at the protein level by co-transfecting 293T cells with the Dies1-3XFlag expression construct in combination with siRNA for either Dies1 or control siRNA. Cell lysates were harvested 48 h later and Dies1 protein determined by Western blot employing an anti-Flag antibody. Figure 5A shows siRNA-mediated knockdown of Dies1 resulted in essentially undetectable levels of the Dies1-Flag protein. The two broad species of Dies1 protein observed by Western blot are attributed to glycosylation events, as previously empirically determined [22]. Figure 5B shows the marked reduction of endogenous Dies1 transcript at 48 h post-transfection of siDies1 into 3T3-L1 adipocytes.

As the siRNA pool targeting Dies1 proved effective, we next determined the effect(s) of Dies1 knockdown on 3T3-L1 adipogenesis. 3T3-L1 preadipocytes were electroporated with siCon, siDies1 or siPPARγ, the latter as positive control. The effectiveness of siPPARγ for knockdown was shown in Figure 4A. Following electroporation to introduce respective siRNAs into 3T3-L1 preadipocytes, cells were plated to attain confluence the next day. After one additional day of culture, preadipocytes were subject to adipogenic induction. Cultures were assessed for lipid accumulation and adipocyte marker gene expression 7 d later. Day 7 adipocytes were photographed live (Figure 5C, upper images) and following fixation and staining with Oil Red O (Figure 5C, lower images). Compared to siCon knocked-down 3T3-L1 adipocytes, Dies1 knocked-down adipocytes evidenced markedly diminished adipocyte differentiation based on lipid accumulation. A representative image of entire culture wells, where neutral lipid is stained with Oil Red O, is shown in Figure 5D. The degree of staining for the siPPARγ treated cultures is essentially background, as Figure 5C and 5D illustrate. To quantify the effect of Dies1 knockdown Oil Red O was extracted and absorbance at 490 nm determined. We also conducted qPCR analysis for a panel of key adipocyte marker transcripts. The bar graphs in Figure 5E, representing two independent knockdown studies, show that siDies1 led to a ∼40% reduction in Oil Red O staining. Figure 5F documents inhibition of adipocyte differentiation for siDies1 cultures at the transcript level. Compared to siCon cultures, a ∼40% to 65% reduction in adipocyte marker transcript levels for PPARγ, stearoyl-coA desaturase 1 (Scd1), adipocyte fatty acid binding protein 4 (Fabp4, also known as aP2), the insulin-responsive solute carrier family 2 facilitated glucose transporter member 4 (Glut4), the adipocyte lipid droplet protein cell death-inducing DFFA-like effector c (Cidec, also known as Fsp27), adipogenin a highly adipocyte-enriched protein (Adig, also known as Smaf1), as well as for Dies1 itself, occurs in siDies1-treated cultures.

Dies1 knockdown has been reported to diminish BMP4 signaling and to enhance expression of pluripotency genes including Oct3/4 and Nanog during *in vitro* ESC differentiation, to inhibit their differentiation [22], [23]. Expression of such genes is generally restricted to pluripotent stem cells. Nonetheless we hypothesized that upregulation of such genes might be a mechanism whereby siDies1 would negatively impact adipogenesis. We found that expression of Oct3/4 and Nanog was essentially undetectable by qPCR for both the siCon and siDies1 3T3-L1 adipocyte cultures (data not shown).

We also conducted knockdown studies to investigate whether Dies1 might play a similar functional role in adipocytes as that described for Dies1 in respect to the differentiation of ESCs, namely enhancement of BMP4 signaling. It had been reported that in the case of ESC differentiation Dies1 was required for effective differentiation and that the mechanism of its action therein was *via* stimulation of BMP4 signaling. In support of this, ESCs knocked down for Dies1 had reduced levels of BMP4 signaling, with siDies1 ESCs showing reduced levels of smad1/5 phosphorylation in response to acute BMP4 stimulation [22]. We determined if a similar decrease in phosho-smad protein level would be observed in 3T3-L1 cells that had been treated with siDies1. This was done by comparing levels of BMP4-induced phospho-smad1 protein in day 7 3T3-L1 adipocytes that were transfected with either siDies1 or siCon. We chose to conduct these studies at this time point since this is when the highest levels of Dies1 are expressed, and therefore when its ability to positively impact BMP4 signaling would likely be most evident. As shown in the western blot in Figure 5G, a robust signal for phospho-smad1 is detected in both siCon and siDies1 cultures following a 15 min exposure to 50 ng/ml BMP4. In contrast to that reported for ESCs, levels of BMP4-induced phospho-smad1 did not decrease when Dies1 was knocked down. This supports the notion that the function of Dies1 in the adipocyte lineage differs from the role of Dies1 in differentiation of ESCs. qPCR analysis also reveals that knockdown of Dies1 during adipogenesis did not lead to statistically meaningful change in levels of BMP4 transcript (Figure 5H). We also find that Dies1 transcript increases during 3T3-L1 adipogenesis, as shown in Figure 1A, but that levels of BMP4 transcript decreases during 3T3-L1 adipogenesis (Figure 5I)). In regard to this, we postulate that if the action of Dies1 in adipogenesis was *via* enhancement of BMP4 signaling, it would be anticipated that BMP4 expression and Dies1 expression in adipogenesis would likely each show the same direction of change. However, instead they show inverse expression, with levels of Dies1 increasing and those for BMP4 decreasing over the course of adipogenesis.

### Enrichment of Dies1 in WAT and Nutritional Regulation

**Figure 6:**
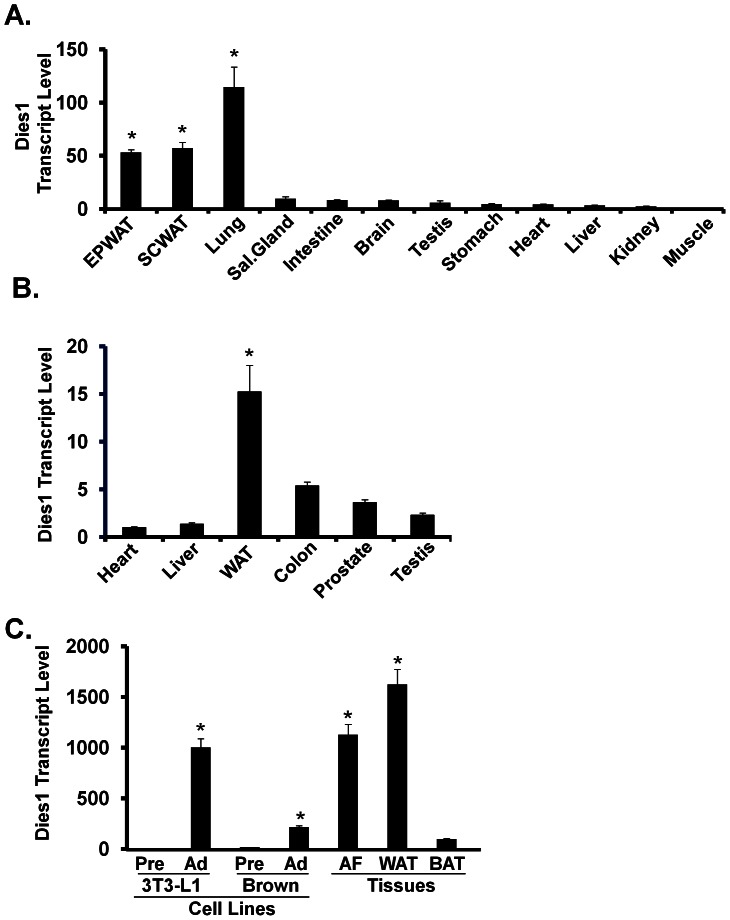
Expression of Dies1 Transcript in Tissues. ***A. Murine Tissues.*** qPCR analysis of Dies1 transcript level in a panel of 12 murine tissues. Value of muscle was set to 1. For first three columns, * indicates *p*<0.05 compared to all other columns. * indicates *p*<0.05 compared to all other columns. ***B. Human Tissues*** **.** qPCR analysis of Dies1 transcript level in a panel of 6 human tissues. Value of heart was set to 1. * indicates *p*<0.05 compared to the columns lacking an asterisk. ***C. Adipocytes and Adipose Tissues.*** qPCR for relative levels of Dies1 transcript in 3T3-L1 or WT-BAT preadipocytes (Pre) or adipocytes (Ad), the purified adipocyte fraction of murine WAT (AF), or intact murine WAT and BAT. Human samples were in monoplicate, murine samples in duplicate; qPCR was conducted in triplicate wells. For each column notated with an* this indicates *p*<0.05 compared to the columns lacking an asterisk.

We next examined distribution of Dies1 transcript across a panel of murine tissues. Figure 6A shows that while the highest site of Dies1 transcript expression is lung, robustly enriched expression for Dies1 is also observed for the two WAT depots examined, epididymal and subcutaneous. Dies1 is also enriched in human WAT in comparison to the five other tissues examined (Figure 6B). Figure 6C compares the relative level of murine Dies1 transcript in white and brown preadipocytes differentiated to adipocytes *in vitro* with that present in white adipocytes obtained directly from WAT and with Dies1 transcript levels in intact WAT or brown adipose tissue (BAT). In regard to WAT, this data shows a similar magnitude of expression of Dies1 transcript in both 3T3-L1 adipocytes and in adipocytes purified directly from WAT. This supports the idea that the 3T3-L1 model is a physiologically relevant setting in which to gain insight into possible regulation and function of Dies1 *in vivo*. In regard to BAT, while Dies1 is found in BAT, its levels therein are much lower than in WAT. Likewise, although the level of Dies1 transcript increased during conversion of brown preadipocytes to adipocytes, both the magnitude of this increase as well as the relative level of Dies1 transcript in differentiated brown adipocytes, is significantly lower than that observed for the 3T3-L1 white adipogenesis *in vitro* model.

**Figure 7:**
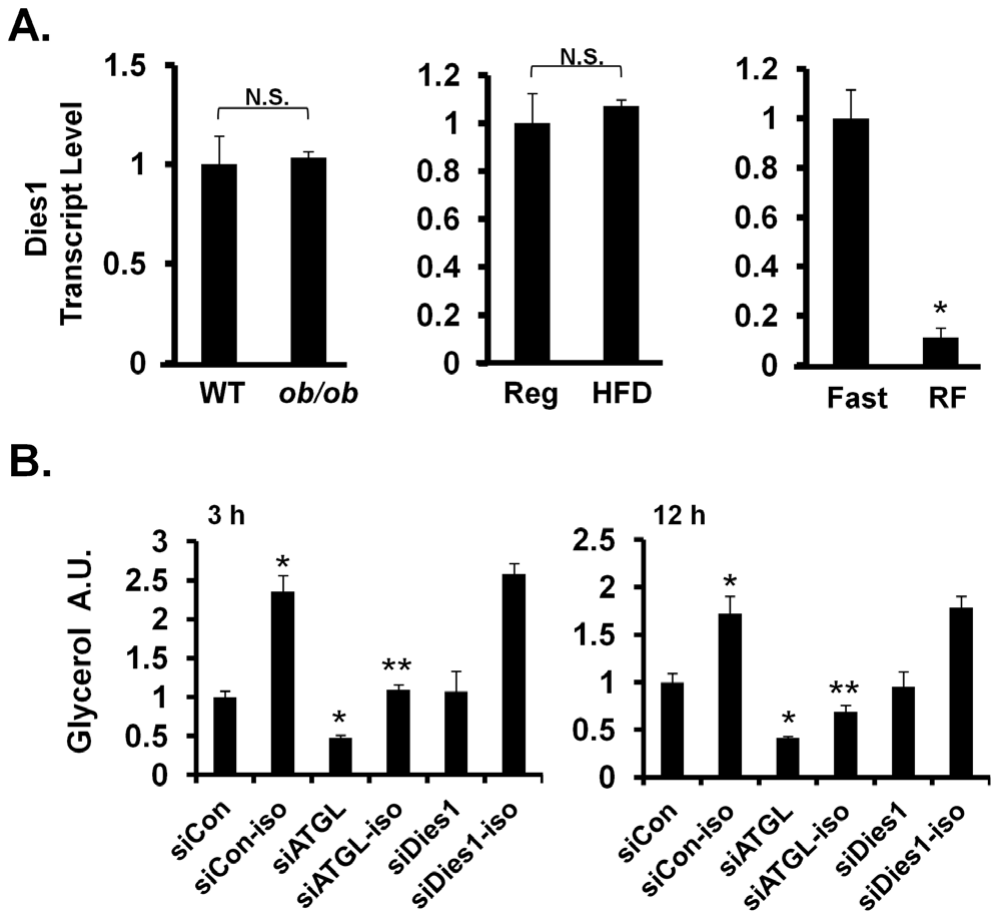
Nutritional Regulation of Dies1 and Lipolysis Assay. ***A. Analysis of Dies1 Transcript in WAT with Obesity and Nutritional Status.*** A. qPCR analysis of Dies1 transcript level in murine WAT in *ob/ob* genetic obesity model (left panel), high fat diet (HFD) *vs.* lean diet (Reg) (middle panel) and in fasted (Fast) and refed murine (RF) WAT (right panel). Value of the first column in each respective graph was set to 1. For WT n = 2; for *ob/ob* n = 2; for Reg n = 3; for HFD, n = 3; for fasted n = 2, and for refed n = 2. ***B. Effect of Dies1 Knockdown on Lipolysis.*** 3T3-L1 adipocytes transfected with the indicated siRNAs were assessed for glycerol release into culture media. Iso indicates isoproterenol treated. For ***A***, * indicates *p*<0.05 compared to the first column of respective graph and N.S. indicates *p*>0.05. For ***B***, * indicates *p*<0.05 compared to first column (siCon) and ** indicates *p*<0.05 compared to second column (siCon-iso). For **7** ***B***, transfections of indicated siRNAs were conducted in at least quadruplicate wells and glycerol assay done in duplicate *per* sample well. A.U., arbitrary units. The overall experiment was conducted twice with essentially the same results; one such study is presented.

The massive induction of Dies1 transcript as precursors convert to mature adipocytes and the highly enriched expression of Dies1 in WAT *in vivo* is consistent with a role for Dies1 in the function of the mature fat cell. To begin to address this we asked if WAT-expressed Dies1 was modulated by metabolic status. To do so, we measured transcript levels in murine WAT in genetic obesity, dietary obesity, and in a fasting and refeeding nutritional regimen. As shown in the two left panels of Figure 7A, neither genetic obesity of the *ob/ob* mouse model, nor high fat dietary (HFD) induced obesity, significantly altered levels of WAT-expressed Dies1. However, we observed a striking 10-fold higher expression of Dies1 transcript in WAT for fasted *vs.* fed mice (Figure 7A, right panel). This acute and marked regulation of Dies1 by nutritional status suggests that this plasma membrane protein may in some manner be involved in the fasting response and/or lipolytic program.

Given higher expression of Dies1 in WAT in the fasted state, we addressed whether Dies1 has a potential regulatory role in lipolysis, utilizing siRNA-mediated knockdown of Dies1 in 3T3-L1 adipocytes. For this study, the non-targeting siRNA, siCon served as negative control. Knockdown of the adipose triglyceride lipase ATGL served as a positive control as ATGL markedly promotes both basal and hormone-stimulated lipolysis [20]. The data for glycerol release is shown in Figure 7B, where the left panel indicates measurements at 3 h and the right panel at 12 h following isoproterenol stimulation. For siCon at the 3 h time point we observe that isoproterenol results in a 2.4-fold increase in free glycerol in culture media compared to untreated; a significant albeit somewhat less of an increase is observed at the 12 h time point. As anticipated, compared to siCon, knockdown of ATGL in adipocytes markedly reduces the level of free glycerol in culture media under basal and isoproterenol stimulated conditions at both the 3 h and the 12 h time point. Data in these same experiments for siDies1 at 3 h and 12 h fail to find an impact of Dies1 knockdown on either basal or isoproterenol stimulated lipolysis.

## Discussion and Conclusions

Here we identify the recently described novel plasma membrane protein Dies1 [22]–[25] as a regulatory factor for adipogenesis. Our observations of upregulation of Dies1 transcript in adipogenesis, but not myogenesis, and its highly selective expression in WAT argue for a degree of selectivity in regard to upregulation and function of Dies1 in the adipocyte lineage, rather than a more general phenomenon of terminal differentiation, *per se*. siRNA studies reveal that reduction of Dies1 during the adipogenesis program results in inhibition of differentiation. This is accompanied by reduced lipid content and reduced expression level of key adipocyte marker transcripts, including that of the master adipogenic regulator PPARγ. We did not detect expression of the brown adipocyte markers uncoupling protein 1 (UCP1) or cell death-inducing DFFA-like effector a (Cidea) in these samples (data not shown), indicating reduction in lipid content was likely not due to attainment of a thermogenic brown adipocyte phenotype. A significant induction of Dies1 during the time course of 3T3-L1 adipogenesis does not occur until day 3, whereas we have consistently observed that a statistically significant increase in PPARγ transcript first occurs at 1 d after adipogenic induction of 3T3-L1 preadipocytes (Figure 1A). This may underlie our observation of a significant but still only partial inhibition of adipogenesis with Dies1 knockdown, since PPARγ is already expressed and presumably driving adipogenesis at the time Dies1 is upregulated at day 3.

As our cellular localization studies show, Dies1 is a plasma membrane protein in adipocytes, and clearly does not localize to lipid droplets. The ectodomain of Dies1 is comprised largely of a single immunoglobulin (IgG) type domain, and its cytoplasmic region is unique. It is currently unknown whether transmembrane Dies1 possesses a signal transduction capability, and there are no clues to such from the sequence of its cytoplasmic region. Its plasma membrane location raises the idea that Dies1 may act in communication between adipocytes and their microenvironment, perhaps by impacting cell shape changes that accompany adipogenic conversion [34]–[36]. Modulation of factors that impact cell shape play a regulatory role in adipogenesis. For example, the MMP2/MT1-MMP axis functions in remodeling of the extracellular environment. This axis is important for adipose tissue expansion that occurs in obesity; mice deficient in MMP2 resist obesity [37]. Studies in null mice indicate that MT-1 MMP is crucial for adipose tissue formation as neonatal MT-1 MMP null mice essentially lack WAT [38]. Dies1 has been reported to impact the function of MMP2 *via* effects on MT-1 MMP. However these studies were conducted with HT1080 fibrosarcoma cells, which produce very high levels of MMP2, and specifically addressed cancer cell growth and invasion [24]. In preliminary studies in this laboratory we investigated if perhaps the role of siDies1 in inhibition of adipogenesis might involve modulation of MMP2 activity. Gelatin zymography was used to compare the levels of pre-pro-MMP2, pro-MMP2 and active MMP2 in 3T3-L1 adipocytes transfected with either siCon or siDies1. We had previously used this method in studies of novel adipokine [39]. However, no reproducible alterations in the levels of any species of MMP2 were observed in siCon *vs.* siDies adipocytes (data not shown).

Dies1 has been demonstrated to promote BMP4 signaling, at least in the single cell line model examined to date, E14Tg2a murine ESCs [22]. However, at this juncture, we believe it is not likely that the inhibition of adipogenesis we observe with siRNA-mediated knockdown of Dies1 is through impinging on BMP4 actions. BMP4 pretreatment, prior to the standard adipogenic induction treatment, can prime mesenchymal stem cells (MSCs) for enhanced differentiation to adipocytes; i.e. converts multipotent stem cells to “committed” preadipocytes. This has been demonstrated for commitment of murine C3H10T1/2 MSCs to a preadipocyte/adipocyte lineage [40]–[42]. It has also been reported that BMP4 promotes preadipocyte commitment and differentiation of human adipose stem cell-like progenitor/stromal cells [43]. However committed preadipocytes such as 3T3-L1 do not need this type of pretreatment to undergo a high degree of adipocyte conversion [18], [19]. The standard Dex, MIX and insulin adipogenic protocol can routinely result in nearly 100% adipogenic conversion [19]. Additionally, if upregulation of Dies1 in adipogenesis were acting *via* enhancement of BMP4 signaling pathways, one might anticipate that Dies1 would be upregulated much earlier in the adipogenic differentiation program. However the upregulation of Dies1 in 3T3-L1 adipogenesis is not seen until day 3, a time point when some cells are already partially converted to adipocytes. It has also been reported that BMP4 transcript levels decrease substantially by day 4 of 3T3-L1 adipogenesis [43] and we have also observed such (Figure 5G); this is the same time frame wherein Dies1 is markedly increasing. If Dies1 were acting to promote BMP4 signaling, as it has been described to do in ESCs, one might rather anticipate a positive correlation between modulation of Dies1 and BMP4 transcript levels during adipogenesis, rather than an inverse one. Moreover, in contrast to the setting of ESCs where Dies1 is expressed and presumably functioning in the precursor cell type, we find that Dies1 expression is minimal in preadipocytes but massively upregulated in differentiated fat cells. Our data also illustrates that levels of BMP4-induced phospho-smad1 protein in 3T3-L1 adipocytes are not reduced when Dies1 is knocked down. At this time, our investigations suggest to us that the action of Dies1 in the adipocyte lineage likely does not involve the ability of Dies1 to enhance BMP4 signaling.

In addition to our siRNA studies that indicate deficiency of Dies1 can inhibit adipogenesis, we also observed that Dies1 expression in murine WAT is strongly regulated during acute nutritional manipulation. In this setting, fasted mice express ∼10 times the level of Dies1 transcript in WAT, compared to levels in the refed state. Induction of WAT Dies1 transcript in times of acute energy demand is also consistent with the lack of alteration in Dies1 transcript expression under long-term conditions of excess WAT, such as we note for *ob/ob* WAT and for WAT of mice on a high fat diet. This fasting-induced increase in WAT Dies1 transcript argues for a role for Dies1 not only in adipogenesis *per se*, but also in mature white adipocytes, perhaps in regulating response to nutritional cues. However, we failed to find an impact of Dies1 knockdown on hormone-stimulated or basal lipolysis. The impact of nutritional regulation of Dies1 on energy metabolism awaits the development of appropriate in *vivo* models.

In conclusion, the highly enriched expression of Dies1 in WAT, its dramatic upregulation during adipogenesis and its acute nutritional regulation *in vivo* indicate a role for this novel transmembrane protein in both formation and function of mature adipocytes.
